# The association of maternal serum parameters with pregnancy induced hypertension in Chinese population

**DOI:** 10.1371/journal.pone.0343337

**Published:** 2026-02-18

**Authors:** Yahui Li, Yun He, Qianqian Wang, Xueni Wang, Yongqiong Xie, Linbo Cheng, Haibo Yao

**Affiliations:** 1 Department of Clinical Laboratory, Chengdu Women’s and Children’s Central Hospital, School of Medicine, University of Electronic Science and Technology of China, Chengdu, China; 2 Department of Obstetrics and Gynaecology, Chengdu Women’s and Children’s Central Hospital, School of Medicine, University of Electronic Science and Technology of China, Chengdu, China; 3 Department of Medical Records, Chengdu Women’s and Children’s Central Hospital, School of Medicine, University of Electronic Science and Technology of China, Chengdu, China; The First Hospital of Jilin University, CHINA

## Abstract

**Background:**

Abnormal changes of peripheral blood parameters were observed in women with pregnancy induced hypertension (PIH) as indicators of multi-organ involvement, but the clinical significance of these promising biomarkers is still debated. This study aimed to determine the role of biochemical parameters and platelet indices for PIH identification and severity evaluation in Chinese population.

**Methods:**

The clinical data of 837 singleton pregnant women were retrospectively analyzed and divided into normotensive control (n = 238), gestational hypertension (n = 213) and preeclampsia (n = 386) groups. Fasting blood samples were collected by first-time examination after admission for measurements by standard techniques. Multivariate logistic regression was performed to assess independent factors correlated with pregnancy induced hypertension. Receiver operating characteristic (ROC) curves were constructed to examine diagnostic accuracy. Correlation analysis was used to evaluate the association between these parameters and Apgar score and birth weight.

**Results:**

The urea, creatinine (Cr), uric acid (UA), aspartate aminotransferase (AST), alanine aminotransferase (ALT), gamma-glutamyl transpeptidase (GGT), lactate dehydrogenase (LDH), mean platelet volume (MPV) and platelet distribution width (PDW) values in the gestational hypertension or preeclampsia groups were significantly higher than those in control group, while total protein (TP), albumin (ALB), prealbumin (PA) and platelet count (PLT) levels were significantly lower. In multivariate regression model, gestational age (GA), body mass index (BMI), UA, ALB, LDH and MPV were found to be independent factors associated with gestational hypertension and preeclampsia. ROC curve analysis indicated that UA, ALB and LDH had good diagnostic performance for preeclampsia with the areas under the curve (AUC) of more than 0.7.

**Conclusion:**

UA, ALB, LDH and MPV have been identified as accessible and valuable biomarkers in diagnosis, severity assessment and prognosis evaluation for gestational hypertension and preeclampsia.

## Introduction

Pregnancy induced hypertension (PIH) is a pregnancy-specific syndrome of unknown etiology, characterized by new-onset hypertension, proteinuria or multisystem dysfunction after the 20th week of pregnancy [1]. Around 5–10% of pregnancies develop PIH worldwide,which is recognized as an important cause of substantial perinatal mortality and morbidity [2,3]. The consequences of this syndrome include the manifestations of maternal multiorgan disorder and the adverse impacts on fetus, e.g., fetal distress, intrauterine growth restriction or even intrauterine death [4,5]. Effective and accurate identification of high-risk women requiring intensive monitoring and prophylactic intervention is crucial to improve perinatal outcomes.

Traditionally, the quantification of proteinuria, reflecting an increased protein excretion due to glomerular endothelial damage, was a most widely recommended parameter for making a diagnosis and evaluating the disease severity [6]. However, a considerable number of studies have shown that the role of proteinuria in progression assessment and adverse outcomes prediction is debatable as some individuals may not present with proteinuria in the clinical course [7,8], in addition, the nonstandard procedures for 24-hour urine sample collection are prone to preanalytical errors, thereby leading to inaccurate results.

So far, biochemical predictors such as AST, ALT, GGT, ALB, Cr, UA and LDH have shown potential values for comprehensive assessment of these disorders as their alterations represent the pathological status of multiorgan dysfunction and exhibit a positive cost-benefit in practical utility [9–11]. A prospective cohort study by Chen et al reported that elevated UA, GGT, and ALP in early gestation are independent risk factors for gestational hypertension or preeclampsia [12]. Corominas et al investigated uric acid levels throughout gestation to propose that uric acid ratio greater than 1.5 may be associated with the onset of preeclampsia [13]. Abnormal changes in platelet indices including PLT, MPV, PDW and PCT have been observed in women diagnosed with PIH and are linked to disease severit [14–16]. The population-based researches suggested that both gestational hypertension and preeclampsia were responsible for a higher risk of poor fetal growth and this impact was even more notable in early-onset cases [17,18].

Pregnancy induced hypertension is a progressive and intractable disorder and the effective treatment is to terminate pregnancy timely. Previous studies have been conducted focusing on the roles of above serum biomarkers in the diagnosis and clinical assessment of preeclampsia, but data of these markers for gestational hypertension is relatively limited. Furthermore, most studies have been undertaken in developed countries, interracial and regional differences on these issues may contribute to divergent results. The aim of the present study was to investigate the association of available peripheral blood parameters in the routine antenatal examination with PIH and perinatal outcomes in Chinese population, and to determine the diagnostic efficacy for those parameters, in order to provide experimental evidence in formulating policies for standard management.

## Materials and methods

### Study design and participants

This retrospective case-control study included 837 pregnant women who received routine antenatal examinations and delivered at the Obstetrics Department of Chengdu Women’s and Children’s Central Hospital, School of Medicine, University of Electronic Science and Technology of China from March 2021 to March 2023. All study participants were singleton pregnancies aged 20–40 years with gestational age above 28 weeks, who had complete medical records and laboratory data. Among them, 599 women with confirmed pregnancy induced hypertension were enrolled into the study group and further stratified into gestational hypertension (n = 213) and preeclampsia (n = 386) groups. Women with a known history of chronic hypertension, hepatic and renal disease, coagulation dysfunction, endocrinologic disorders, infectious etiologies and those with other diseases or on anticoagulant/antiplatelet drugs such as aspirin and heparin that may affect the levels of evaluated peripheral blood variables were excluded from the study. Furthermore, research subjects complicated with diabetes mellitus, intrahepatic cholestasis, reproductive infections, recurrent miscarriage and assisted reproduction were also excluded from this analysis. Another 238 normotensive pregnant women without underlying maternal condition during the same period were randomly selected as controls group.

### Definition of gestational hypertension and preeclampsia

The diagnostic criteria for pregnancy induced hypertension referred to Chinese guideline for diagnosis and treatment of hypertensive disorders in pregnancy (2015 Edition) [19]. Gestational hypertension (GH) was defined as blood pressure ≥140/90 mmHg measured at least twice (4 hours apart) in previously normotensive women after 20 weeks of pregnancy, without proteinuria or a sign of multiorgan involvement, severe gestational hypertension was diagnosed as continuous developed blood pressure level ≥ 160/110 mmHg. Preeclampsia (PE) was defined as newly occurring hypertension accompanied with significant proteinuria (≥0.3g/24hours, protein/creatinine ratio≥0.3 or strip test≥1+) or in the absence of proteinuria but combined with the evidence of multisystem disorder. Preeclampsia patients with any of the following condition was regarded severe cases: severe hypertension (blood pressure≥160/110 mmHg), liver dysfunction (elevated transaminase twice the norm), impaired renal function (proteinuria ≥ 2g/24hours, oliguria, creatinine>106μmol/L), coagulopathy, cerebral or visual disturbances, pulmonary edema, uteroplacental dysfunction and so on. Early-onset preeclampsia was case occurring before 34 weeks of gestation and late-onset preeclampsia was case occurring after 34 weeks of gestation.

### Data collection and measurements

Data collection was conducted on march 10, 2025 after the approval was obtained from the ethics committee on march 8, 2025. Detailed clinical features at the time of admission prior to delivery (age, height, weight, nulliparity, systolic blood pressure, diastolic blood pressure, gestational age), laboratory findings, mode of delivery, adverse pregnancy outcomes, apgar scores and birth weight were obtained from the electronic medical record of each patient. Body mass index (BMI) was calculated as weight (kg) divided by the square of height (m).

Fasting blood samples were collected in vacutainer tubes for the first time measurement after hospitalization and detected within 2 hours. Biochemical testing was performed by immunoturbidimetry assay using Hitachi 7600 automatic analyzer (Hitachi Corporation, Tokyo, Japan). Whole blood counts were measured by Sysmex XN-1000 hematology analyzer (Sysmex Corporation, Kobe, Japan). Total volume of 24h urine sample (from 8 am to 8 am the next day) was accurately recorded for proteinuria assay using Hitachi 7600 automatic biochemical analyzer (Hitachi Corporation, Tokyo, Japan). All operations are conducted strictly in accordance with the manufacturer’s instructions.

### Statistical analysis

All statistical analyses were conducted with SPSS 23.0 software (SPSS Inc., Chicago, IL, USA). Based on the normality of the data, continuous variables were represented as mean and standard deviation or median and interquartile range, the differences between two groups were analyzed by the independent-samples t test or Mann–Whitney U-test, and multiple comparisons were performed using one-way ANOVA or Kruskal-Wallis test. The categorical data were described in the form of frequency (percentage) and compared by Chi-square test or Fisher exact test. To assess the association of maternal serum parameters with PIH prevalence in pregnant women, multivariate logistic regression models were performed by calculating the odds ratio(OR) with 95% confidence interval(CI) or P value. Receiver operating characteristic (ROC) curves were constructed to determine the diagnostic ability of evaluated markers, the area under the curve (AUC), cut-off values, sensitivity, and specificity were obtained. Spearman correlation coefficient was used to investigate the correlation between individual continuous variables. P-values <0.05 were considered to be statistically significant.

### Ethics statement

This study was conducted after approval was obtained from the ethics committee of Chengdu Women’s and Children’s Central Hospital (Lot No.: 2025(38)). The management of patient data in this research strictly conformed to the principles of the Helsinki Declaration, confidentiality and anonymity was maintained throughout the study. As it was a retrospective study, the ethics committee waived the requirement for informed consent.

## Results

### Clinical characteristics and laboratory variables of study participants

A total of 837 eligible pregnant women were enrolled in the study, among these, 238 were normotensive pregnancies, 213 had gestational hypertension and 386 had preeclampsia. The clinical characteristics and laboratory findings of all participants are summarized in Table 1. The median age of the control, gestational hypertension and preeclampsia group was 29.00(27.00–31.00), 29.00(27.00–32.00), and 30.00 (27.00–32.00) respectively. The median parity of the control, gestational hypertension and preeclampsia group was 0.00(0.00,1.00), 0.00(0.00,1.00), 0.00(0.00,1.00). There were no significant differences in age, gravidity and parity between these groups. When compared with those in control group, the body mass index (BMI), systolic blood pressure, diastolic blood pressure and cesarean rate were significantly higher, while gestational age at delivery, birth weight and Apgar scores at 1 minute trended lower in GH and PE groups, especially in the PE group. Additionally, the incidences of preterm birth, intrauterine growth restriction, neonatal asphyxia were found higher in the PE group than those in the control group, although no significant differences in these parameters was observed between the control and GH groups. Laboratory parameters including Urea, Cr, UA, ALT, AST, GGT, TP, ALB, PA, LDH, PLT, MPV and PDW showed a statistical difference in the univariate analysis across all three groups (p < 0.05). The GH and PE group had significantly higher levels of Urea, UA, ALT, GGT, MPV and PDW than that of control group, along with significantly lower ALB and PA levels, moreover, significant differences in the values of Urea, UA, ALT, ALB exist between the GH and PE group. In comparison with controls, the Cr, AST and LDH values were higher and TP and PLT values were lower in PE group, while there were no significant differences by comparing the five variable**s** between GH and control group. No statistically significant differences in alkaline phosphatase (ALP), total bile acid (TBA), and plateletcrit (PCT) values were observed between three groups.

**Table 1:** The clinical characteristics and laboratory markers of the normotensive controls, gestational hypertension and preeclampsia cases.

	Control (n = 238)	GH (n = 213)	PE (n = 386)	p value	P *	P**	P***
**Maternal**							
Age (years)	29.00(27.00,31.00)	29.00(27.00,32.00)	30.00(27.00,32.00)	0.063			
BMI (kg/m^2^)	26.56(24.77,28.24)	27.89(25.97,29.97)	28.53(26.21,30.62)	<0.001	<0.001	<0.001	
Gravidity (times)	2.00(1.00,2.00)	2.00(1.00,3.00)	2.00(1.00,2.00)	0.934			
Parity (times)	0.00(0.00,1.00)	0.00(0.00,1.00)	0.00(0.00,1.00)	0.053			
Systolic blood pressure (mmHg)	114.00(108.00,120.00)	131.00(124.00,140.00)	138.00(130.00,146.25)	<0.001	<0.001	<0.001	<0.001
Diastolic blood pressure (mmHg)	74.00(70.00,79.00)	90.00(84.00,95.00)	93.00(87.00,100.25)	<0.001	<0.001	<0.001	0.001
Gestational age at deliver (weeks)	39.00(38.00,40.00)	39.00(38.00,39.00)	38.00(36.00,39.00)	<0.001	<0.001	<0.001	<0.001
Vaginal delivery (%)	150(63)	84(39.4)	86(22.3)	<0.001	<0.05	<0.05	<0.05
Caesarean section (%)	88 (37)	129 (60.6)	300 (77.7)				
HELLP syndrome (%)	0(0.0%)	0(0.0%)	6(1.6%)	0.041			
Eclampsia (%)	0(0.0%)	0(0.0%)	3(0.8%)	0.343			
**Laboratory parameters**							
Gestational age at sampling (weeks)	39.00(38.00,39.00)	38.00(37.00,39.00)	38.00(36.00,39.00)	<0.001	<0.001	<0.001	0.012
Urea (mmol/L)	3.23(2.81,3.98)	3.68(3.03,4.46)	4.04(3.33,5.00)	<0.001	0.001	<0.001	0.002
Cr (µmol/L)	43.50(38.45,49.20)	44.10(40.40,50.90)	50.00(42.58,58.40)	<0.001		<0.001	<0.001
UA (µmol/L)	299.20(261.20,335.00)	348.50(294.00,407.00)	400.30(339.75,487.53)	<0.001	<0.001	<0.001	<0.001
ALT (U/L)	12.20(9.00,16.85)	14.00(11.30,19.70)	17.55(12.90,27.00)	<0.001	0.001	<0.001	<0.001
AST (U/L)	18.60(15.90,21.85)	18.90(15.10,23.10)	21.35(17.08,28.53)	<0.001		<0.001	<0.001
GGT (U/L)	12.30(9.05,19.05)	14.50(10.50,25.60)	17.55(10.98,30.00)	<0.001	0.033	<0.001	
ALP (U/L)	165.20(139.45,207.45)	160.80(131.60,206.70)	161.25(122.38,195.18)	0.188			
TP (g/L)	63.65 ± 3.80	63.52 ± 4.49	60.60 ± 5.59	<0.001		<0.001	<0.001
ALB (g/L)	36.30(34.75,37.60)	35.10(33.30,37.00)	33.45(30.60,35.70)	<0.001	0.002	<0.001	<0.001
PA (mg/L)	214.00(196.45,236.15)	215.70(193.90,236.00)	197.05(168.08,226.13)	<0.001	<0.001	<0.001	
TBA (µmol/L)	3.50(2.50,5.10)	3.60(2.40,5.50)	3.50(2.20,5.73)	0.392			
LDH (U/L)	176.00(157.75,195.30)	184.00(164.00,205.80)	205.00(181.85,235.65)	<0.001		<0.001	<0.001
PLT (10^9^/L)	185.00(156.50,231.50)	184.00(154.00,225.00)	177.00(141.75,224.00)	0.004		0.007	
MPV (fl)	11.40(10.70,12.45)	11.90(11.00,12.80)	12.10(10.98,13.00)	<0.001	0.002	<0.001	
PDW (fl)	14.10(12.50,17.15)	15.60(12.90,18.60)	15.90(13.20,19.23)	<0.001	0.009	<0.001	
PCT (%)	0.22(0.19,0.27)	0.22(0.19,0.26)	0.22(0.18,0.25)	0.163			
Proteinuria (g/24 h)	—	0.10(0.15,0.23)	0.50(1.34,2.21)	<0.001			
**Neonatal**							
Preterm birth < 37 week) (%)	5(2.1%)	14(6.6%)	100(25.9%)	<0.001		<0.05	<0.05
Preterm birth < 34 weeks (%)	1(0.4%)	5(2.3%)	54(14.0%)	<0.001		<0.05	<0.05
Intrauterine growth restriction (%)	4(1.7%)	8(3.8%)	59(15.3%)	<0.001		<0.05	<0.05
Low birth weight (%)	5(2.1%)	19(8.9%)	99(25.6%)	<0.001	<0.05	<0.05	<0.05
Neonatal asphyxia (%)	1(0.4%)	7(3.3%)	23(6.0%)	0.002		<0.05	
Stillbirth (%)	0(0.0%)	0(0.0%)	7(1.8%)	0.020			
Apgar score at 1 min	10.00(10.00,10.00)	10.00(9.00,10.00)	10.00(9.00,10.00)	<0.001	<0.001	<0.001	
Apgar score at 5 min	10.00(10.00,10.00)	10.00(10.00,10.00)	10.00(10.00,10.00)	<0.001		<0.001	<0.001
Birth weight (g)	3310.00(3100.00,3580.00)	3170.00(2950.00,3500.00)	3035.00(2467.50,3380.00)	<0.001	<0.001	<0.001	0.001

Non-normally distributed data are expressed as median (interquartile range), normally distributed data are expressed as mean±standard deviation.

GH: gestational hypertension PE: preeclampsia

p value Comparison among groups with control, GH and PE

P * Comparison between control and GH

P** Comparison between control and PE

P*** Comparison between GH and PE

### Comparison of preeclampsia subcategories

The preeclampsia (PE) group was further stratified into different subgroups according to severity and onset time, the comparisons between the preeclampsia subcategories are presented in Table 2. Various clinical parameters were compared between women with mild preeclampsia and severe preeclampsia, the rates of caesarean section, HELLP syndrome, preterm birth, intrauterine growth restriction, neonatal asphyxia, stillbirth, birth weight, Apgar scores and gestational age at delivery were significantly different. The Urea, Cr, UA, ALT, AST, LDH and proteinuria levels were found to be higher as the disease progressed, whereas ALP, TP, ALB, PA, PLT, PCT values were significantly decreased. Between early-onset and late-onset preeclampsia cases, perinatal complications such as HELLP syndrome, premature, intrauterine growth restriction, neonatal asphyxia and stillbirth were more frequent in the early-onset preeclampsia group, along with lower birth weight, Apgar scores at 1 and 5 min and gestational age at delivery, laboratory findings showed significantly higher Urea, Cr, UA, ALT, AST, GGT, LDH values and lower ALP, TP, ALB, PA levels were observed in the early-onset group in comparison to the late-onset group. Maternal general characteristics for age, body mass index, gravidity and parity were similar between the subgroups.

**Table 2:** Comparison of preeclampsia subcategories.

	Severity		P	Onset time		P
	Mild (n = 218)	Severe (n = 168)		Early onset (n = 71)	Late onset (n = 315)	
**Maternal**						
Age (years)	29.00(27.00,32.00)	30.00(27.00,32.75)	0.327	30.14 ± 4.48	29.66 ± 3.89	0.362
BMI (kg/m^2^)	28.46 ± 3.06	28.70 ± 3.30	0.457	28.81 ± 3.50	28.51 ± 3.08	0.472
Gravidity (times)	1.00(1.00,2.00)	2.00(1.00,3.00)	0.583	2.00(1.00,3.00)	1.00(1.00,2.00)	0.323
Parity (times)	0.00(0.00,1.00)	0.00(0.00,1.00)	0.826	0.00(0.00,1.00)	0.00(0.00,1.00)	0.665
Systolic blood pressure (mmHg)	133.32 ± 11.88	145.33 ± 15.92	<0.001	144.00(134.00,153.00)	136.00(128.00,146.00)	<0.001
Diastolic blood pressure (mmHg)	91.00(84.75,96.00)	98.00(90.00,105.75)	<0.001	99.00(90.00,106.00)	92.00(86.00,99.00)	<0.001
Gestational age at deliver (weeks)	38.00(38.00,39.00)	37.00(34.00,38.00)	<0.001	33.00(30.00,34.00)	38.00(37.00,39.00)	<0.001
Vaginal delivery (%)	59(27.1)	27(16.1)	0.010	8(11.3)	78(24.8)	0.014
Caesarean section (%)	159(72.9)	141(83.9)		63(88.7)	237(75.2)	
HELLP syndrome (%)	0(0.0%)	6(3.6%)	0.006	4(5.6%)	2(0.6%)	0.012
Eclampsia (%)	0(0.0%)	3(1.8%)	0.082	2(2.8%)	1(0.3%)	0.088
**Laboratory parameters**						
Gestational age at sampling (weeks)	38.00(37.00,39.00)	37.00(33.00,38.00)	<0.001	33.00(30.00,34.00)	38.00(37.00,39.00)	<0.001
Urea (mmol/L)	3.90(3.21,4.71)	4.40(3.43,5.42)	<0.001	4.73(3.48,5.93)	3.94(3.33,4.88)	0.001
Cr (µmol/L)	46.60(40.78,54.75)	54.05(46.48,62.85)	<0.001	56.00(46.70,64.50)	48.80(41.70,56.60)	0.001
UA (µmol/L)	380.95(322.40,456.58)	440.65(377.58,506.95)	<0.001	449.00(370.50,533.40)	394.20(333.10,475.00)	0.002
ALT (U/L)	16.50(12.68,24.93)	20.05(13.90,34.75)	0.015	23.40(15.90,47.20)	16.60(12.50,25.60)	0.001
AST (U/L)	20.55(16.50,26.35)	23.80(18.73,31.15)	0.009	25.70(18.50,42.00)	21.00(17.00,27.40)	0.002
GGT (U/L)	17.60(11.00,27.10)	17.00(10.40,33.33)	0.338	27.90(12.80,56.20)	16.50(10.50,26.00)	<0.001
ALP (U/L)	169.45(132.55,202.85)	151.90(115.10,186.98)	0.003	139.30(108.00,183.50)	165.00(128.00,202.80)	0.009
TP (g/L)	62.19 ± 4.58	58.53 ± 6.11	<0.001	57.96 ± 5.65	61.19 ± 5.41	<0.001
ALB (g/L)	34.55(32.60,36.20)	31.50(28.50,34.20)	<0.001	31.00(28.10,33.30)	34.00(31.50,35.80)	<0.001
PA (mg/L)	210.10(175.65,230.53)	187.30(158.43,209.53)	<0.001	183.69 ± 39.03	198.36 ± 38.18	0.004
TBA (µmol/L)	3.50(2.40,5.63)	3.50(1.90,6.00)	0.155	2.70(1.60,6.30)	3.60(2.40,5.80)	0.064
LDH (U/L)	197.20(179.00,225.00)	216.05(190.18,271.88)	<0.001	231.50(196.30,271.80)	202.30(180.05,228.00)	<0.001
PLT (10^9^/L)	176.50(146.75,220.25)	169.50(129.00,218.50)	0.044	174.00(132.00,240.00)	173.00(140.00,215.00)	0.291
MPV (fl)	12.15(11.00,13.00)	12.20(11.00,13.30)	0.540	11.80(10.90,13.00)	12.20(11.00,13.10)	0.338
PDW (fl)	16.15(13.48,19.20)	15.70(13.20,20.08)	0.236	15.10(12.90,18.80)	16.20(13.40,20.00)	0.180
PCT (%)	0.22(0.19,0.26)	0.21(0.17,0.25)	0.041	0.21(0.17,0.28)	0.21(0.18,0.25)	0.401
Proteinuria (g/24 h)	0.90(0.48,1.67)	1.98(0.71,3.47)	<0.001	1.54(0.52,2.93)	1.34(0.50,2.09)	0.221
**Neonatal**						
Preterm birth < 37 week) (%)	21(9.6%)	79(47.0%)	<0.001	70(98.6%)	30(9.5%)	<0.001
Preterm birth < 34 weeks (%)	8(3.7%)	46(27.4%)	<0.001	54(76.1%)	0(0.0%)	<0.001
Intrauterine growth restriction (%)	12(5.5%)	47(28.0%)	<0.001	37(52.1%)	22(7.0%)	<0.001
Low birth weight (%)	21(9.6%)	78(46.4%)	<0.001	63(88.7%)	36(11.4%)	<0.001
Neonatal asphyxia (%)	3(1.4%)	20(11.9%)	<0.001	19(26.8%)	4(1.3%)	<0.001
Stillbirth (%)	0(0.0%)	7(4.4%)	0.003	7(9.9%)	0(0.0%)	<0.001
Apgar score 1 min	10.00(9.00,10.00)	9.00(9.00,10.00)	<0.001	8.00(8.00,9.00)	10.00(9.00,10.00)	<0.001
Apgar score 5 min	10.00(10.00,10.00)	10.00(9.00,10.00)	<0.001	9.00(9.00,10.00)	10.00(10.00,10.00)	<0.001
Birth weight (g)	3200.00(2890.00,3542.50)	2625.00(1977.50,3100.00)	<0.001	1700.00(1300.00,2050.00)	3120.00(2800.00,3460.00)	<0.001

Non-normally distributed data are expressed as median (interquartile range), normally distributed data are expressed as mean±standard deviation.

### Multivariate logistic regression analysis of factors associated with pregnancy induced hypertension

In order to further examine the potential influential variables associated with gestational hypertension and preeclampsia, the identified factors with statistical significance in the univariate analysis were enrolled into multivariate logistic regression analysis. As shown in Table 3, gestational age, BMI, UA, ALB, LDH and MPV were found to be independent factors associated with GH (gestational age: OR=0.607, 95% CI = 0.496–0.743; BMI: OR=1.195, 95% CI = 1.099–1.300; UA: OR=1.009, 95% CI = 1.005–1.012; ALB: OR=0.905, 95% CI = 0.822–0.996;LDH: OR=1.008, 95% CI = 1.001–1.016; MPV: OR=1.245, 95% CI = 1.028–1.509) and PE (gestational age: OR=0.495, 95% CI = 0.403–0.608; BMI: OR=1.268, 95% CI = 1.163–1.383; UA: OR=1.012, 95% CI = 1.008–1.016; ALB: OR= 0.814, 95% CI = 0.738–0.898;LDH: OR=1.019, 95% CI = 1.011–1.027; MPV: OR=1.272, 95% CI = 1.042–1.552).

**Table 3:** Multivariate logistic regression model of factors associated with gestational hypertension and preeclampsia.

	B	Standard error	WALD	df	Sig.	OR (95 CI%)
**GH**						
Intercept	11.639	4.515	6.646	1	0.010	
Age	−0.029	0.032	0.794	1	0.373	0.971 (0.912-1.035)
Gestational age	−0.499	0.103	23.311	1	0.000	0.607 (0.496-0.743)
BMI	0.178	0.043	17.185	1	0.000	1.195 (1.099-1.300)
Urea	0.130	0.120	1.174	1	0.279	1.139 (0.900-1.441)
UA	0.009	0.002	22.168	1	0.000	1.009 (1.005-1.012)
AST	−0.025	0.015	2.765	1	0.096	0.975 (0.947-1.005)
GGT	0.013	0.010	1.794	1	0.180	1.013 (0.994-1.033)
ALB	−0.100	0.049	4.157	1	0.041	0.905 (0.822-0.996)
LDH	0.008	0.004	4.473	1	0.034	1.008 (1.001-1.016)
MPV	0.219	0.098	5.014	1	0.025	1.245 (1.028-1.509)
**PE**						
Intercept	16.928	4.567	13.736	1	0.000	
Age	0.006	0.033	0.032	1	0.857	1.006 (0.943-1.074)
Gestational age	−0.703	−0.105	44.61	1	0.000	0.495 (0.403-0.608)
BMI	0.238	0.044	28.735	1	0.000	1.268 (1.163-1.383)
Urea	0.215	0.123	3.034	1	0.082	1.240 (0.973-1.578)
UA	0.012	0.002	39.097	1	0.000	1.012 (1.008-1.016)
AST	−0.021	0.014	2.147	1	0.143	0.979 (0.952-1.007)
GGT	0.018	0.010	3.459	1	0.063	1.018 (0.999-1.038)
ALB	−0.206	0.050	16.956	1	0.000	0.814 (0.738-0.898)
LDH	0.019	0.004	22.122	1	0.000	1.019 (1.011-1.027)
MPV	0.240	0.102	5.606	1	0.018	1.272 (1.042-1.552)

GH: gestational hypertension PE: preeclampsia

### Diagnostic performance of significant markers for gestational hypertension and preeclampsia

**Fig 1:**
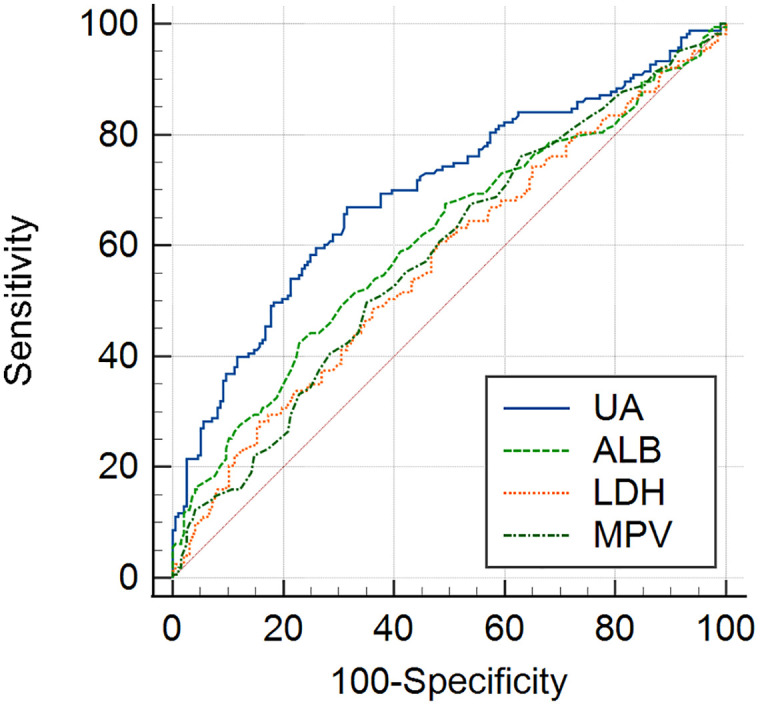
Receiver operating characteristic (ROC) curve of UA, ALB, LDH and MPV in pregnant women with GH.

**Fig 2:**
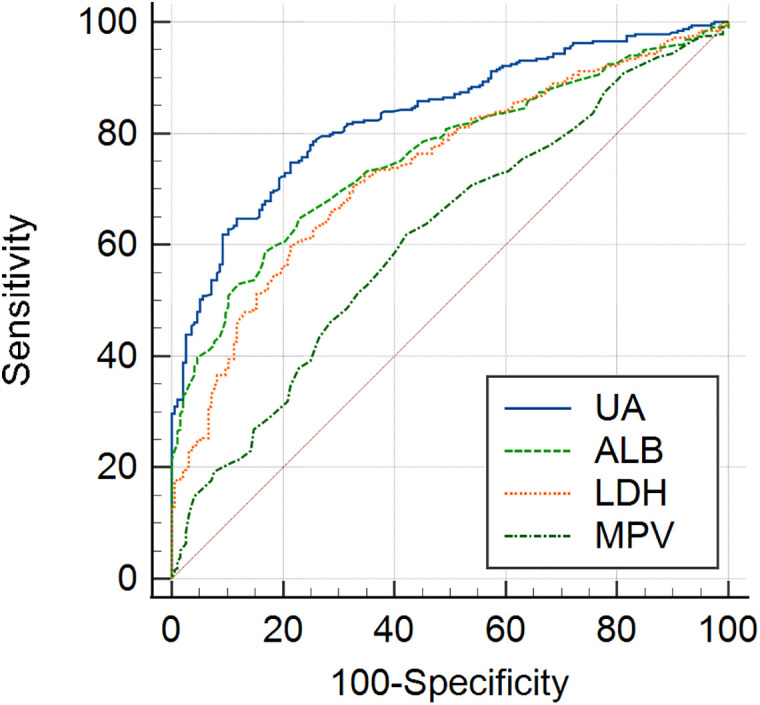
Receiver operating characteristic (ROC) curve of UA, ALB, LDH and MPV in pregnant women with PE.

The diagnostic efficacy of UA, ALB, LDH and MPV as potential biomarkers for pregnancy induced hypertension was subsequently determined by ROC analysis (Table 4). UA had an area under the curve (AUC) of 0.707 with 68.54% sensitivity and 67.65% specificity and were considered as appropriate marker for GH. Although ALB, LDH and MPV were identified as significant factors by multivariate logistic analysis, ROC curves of these parameters showed low diagnostic values to distinguish GH patients and normotensive pregnancy (AUC < 0.7) (Fig 1). We found that UA which had the highest AUC of 0.833, along with sensitivity of 65.03% and specificity of 88.66% showed a good diagnostic performance in identifying PE from controls, followed by ALB (AUC = 0.745, sensitivity = 56.99%, specificity = 81.09%) and LDH (AUC = 0.737, sensitivity = 59.62%, specificity = 78.68%), while MPV showed a weak diagnostic efficacy for PE (AUC < 0.70) (Fig 2).

**Table 4:** Diagnostic efficacy of UA, ALB, LDH and MPV as markers for gestational hypertension and preeclampsia.

	AUC (95% CI)	P value	Cutoff value	Sensitivity	Specificity	Youden index
**GH**						
UA	0.707 (0.663-0.749)	< 0.001	319.9	68.54%	67.65%	0.362
ALB	0.601 (0.555-0.647)	< 0.001	34.7	45.07%	72.27%	0.173
LDH	0.570 (0.518-0.622)	0.021	177.8	60.74%	51.78%	0.125
MPV	0.592 (0.545-0.638)	< 0.001	11.9	49.77%	65.97%	0.157
**PE**						
UA	0.833 (0.802-0.862)	< 0.001	367.7	65.03%	88.66%	0.537
ALB	0.745 (0.709-0.779)	< 0.001	34.10	56.99%	81.09%	0.381
LDH	0.737 (0.697-0.774)	< 0.001	197.4	59.62%	78.68%	0.383
MPV	0.631 (0.592-0.669)	< 0.001	11.60	63.47%	58.40%	0.219

GH: gestational hypertension PE: preeclampsia

### Correlation analysis of laboratory markers with Apgar score and birth weight in pregnancy induced hypertension

In this study, association of these significant parameters with birth weight and apgar score in PIH patients was evaluated by the Spearman correlation analysis. As shown in Table 5, maternal serum UA and LDH levels were negatively associated with neonatal apgar score 1 min, apgar score 5 min and birth weight. On the other hand, the ALB levels showed significant positive correlation with birth weight, apgar score 1 min and apgar score 5 min. MPV showed no statistically significant correlation with birth weight and apgar score.

**Table 5:** Association between laboratory markers in women with pregnancy induced hypertension and Apgar score and birth weight.

	Birth weight		Apgar score 1 min		Apgar score 5 min	
	r	p	r	p	r	p
UA	−0.203**	0.000	−0.155**	0.000	−0.136**	0.000
ALB	0.186**	0.000	0.118**	0.001	0.212**	0.000
LDH	−0.145**	0.000	−0.080*	0.038	−0.188**	0.000
MPV	0.007	0.841	−0.063	0.070	−0.037	0.284

**p < 0.01, * p < 0.05

## Discussion

Pregnancy induced hypertension (PIH), a multisystemic and progressive disorder, includes gestational hypertension and preeclampsia, and remains a major problem of maternal and fetal health worldwide. Although the outcomes of gestational hypertension are normally good, women with gestational hypertension occurring before 34 gestational weeks may progress into preeclampsia resulting in severe perinatal complications [20], so early diagnosis and adequate assessment of disease severity would facilitate effective management and intervention. The exact mechanisms of PIH have not been fully understood, the clinical diagnosis was based on new-onset high blood pressure or proteinuria. Blood pressure measurement is susceptible to interference factors such as patient status, measurement time, position, and equipment. Proteinuria usually appears after renal dysfunction and correlates poorly with maternal–fetal outcomes**.** In addition, high blood pressure and proteinuria is neither specific nor sensitive enough for diagnosis. In recent years, angiogenic factors, placental growth factor (PIGF), C‑reactive protein (CRP), interleukin‑6 (IL‑6), endothelial cell-specific molecular molecule-1 (ESM-1) have been proposed to be predictors for preeclampsia, but the clinical value of the above markers are still under investigation and their utilization as routine screening procedures is questionable in resource-limited areas. Widely accessibile and cost-effective peripheral blood indices of routinely performed examination characterizing the degree of multiorgan damage can be effective monitoring indicators to assess the progression of pregnancy-induced hypertension [21,22]. In this work, we found that abnormal changes in peripheral blood variables (ALT, AST, GGT, ALP, TP, ALB, PA, LDH, Urea, Cr, UA, PLT, MPV, PDW) were closely related to the onset and severity of pregnancy-induced hypertension, GA, BMI, UA, ALB, LDH and MPV were independent factors associated with gestational hypertension and preeclampsia in multivariable analyses.

Serum ALT, AST, GGT, ALP, TP, ALB, PA, TBA and LDH are known markers for liver dysfunction. Previous studies showed that elevated ALT, AST, GGT, ALP and TBA levels were positively associated with new-onset hypertension during pregnancy [23–26]. Similar to above studies, we found serum ALT, AST and GGT levels in women with gestational hypertension or preeclampsia was significantly higher than that in normal pregnancie, since hypoxia and systemic inflammatory response followed by hypoperfusion may contribute to degeneration and necrosis of hepatocytes and release of liver enzymes [27]. But no significant elevation in ALP and TBA values were observed among women diagnosed with PIH in this study, which is consistent with Rahman et al’ s study [28]. The disagreement could probably be due to variations in the gestational week or exclusion of patients complicated with intrahepatic cholestasis of pregnancy (ICP). Although AST, ALT, GGT, TP and PA levels signifcantly changed among hypertensive pregnancie, none of these showed significant association with gestational hypertension and preeclampsia in multivariable analyses.

LDH is an intracellular enzyme reflecting cellular damage and dysfunction in a state of hypoxia [29]. It has also been proposed as a good predictor of PE severity and an indicator of multiorgan involvemen [30]. We observed that the LDH value of PE group was also higher than that of control group, and there was significant difference between GH group and PE group, suggesting higher LDH value might have potential values in distinguish patients with PE and GH. Our finding demonstrated that the increasing level of LDH was an independent risk factor for pregnancy-induced hypertension and the AUC for serum LDH to identify preeclampsia was 0.737, with higher specificity in comparison to other liver enzymes. Furthermore, elevated LDH levels had significant correlation with birth weight and apgar score to show adverse outcomes associated with preeclampsia.

Dai et al reported that the ALB values of women with preeclampsia and eclampsia during the third trimester were lower than those of normal pregnancies [31]. Moreover, this study showed that the levels of ALB and PA in either gestational hypertension or preeclampsia group were lower compared to control group, it might be a consequence of plasma proteins loss and synthetic function impairment [32]. From the ROC curve analysis, the results suggested that ALB levels might be a useful marker of reasonably good sensitivity and high specificity for preeclampsia diagnosis. Furthermore, pregnant women with severe proteinuria are prone to hypoproteinemia, which may be closely related to disease severity and adverse perinatal complications.

When renal arterioles spasm induced by hypertension results in the decrease of renal blood perfusion, abnormal function indexes can be detected for monitoring the extent of kidney injury. In the current study, a signifcant elevation in serum urea, creatinine, and uric acid levels was observed among women with pregnancy-induced hypertension, similar results were reported by Ekun et al [33]. Also, our study revealed that serum UA was an important independent factor associated with GH and PE, Corominas et al noted that the elevated uric acid levels precedes the development of hypertension and proteinuria in PE [34]. Based on ROC curves, UA level had the largest AUC and a superior diagnostic accuracy among those markers to differentiate the severity of pregnancy-induced hypertension. This indicates that UA level is more sensitive and reliable than urea and creatinine in reflecting the renal damage progression in patients with PIH. Therefore, Besides known reduced renal excretion, an antioxidant effect on endothelium and the interaction with inflammation may be other contributing factors of the increased uric acid levels observed in pregnancy-induced hypertension [35]. As observed in study of Ryu et al [36], our results showed maternal serum uric acid level prior to delivery related inversely to neonatal birth weight and apgar score.

In pregnancy-induced hypertension, increased platelet activation due to endothelial damage induced platelet aggregation and consumption, which may result in platelet variance in number, volume and activity [37]. Bawore et al found an increase of MPV and PDW value and a decrease in PLT and PCT level in PE patients and the differences were statistically significant [38]. Lin et al reported that increased PLT and PCT during early pregnancy were at higher risk of developing GH and PE, while higher MPV was associated with GH in propensity score analysis [39]. This discrepancy could be explained by the variation in the sample collection time and retention time. In the current study, the MPV and PDW levels in GH and PE groups were significantly higher compared to those in the control group and PLT was found significantly decreased in the PE group. Moreover, MPV value was an independent factor for PIH, indicating that MPV may be a more responsive marker than PLT and PDW to define the enhancement of platelet activity during PIH progression and platelet indices should be monitored throughout pregnancy.

Pregnancy induced hypertension (PIH) was reported to have a higher risk of low birth weight and neonatal asphyxia, which was consistent with the neonatal birth weight and Apgar score. The results of correlation test showed that UA, ALB and LDH were signifcantly correlated with birth weight and Apgar score among PIH pregnancies in this study, which may be attributed to the artificial manipulation for severe cases needing to be terminated immediately. Therefore, further large cohort studies are required to understand the associations with perinatal outcomes affected by multiple factors and establish predictive threshold values for individual or composite adverse outcomes in clinical management.

The hematological parameters are primary screening indexes for antenatal investigation with advantages of simplicity and feasibility, this study investigated the independent factors for the determination of PIH with exclusion of participants with potential confounders which might affect the accuracy of the results. Notably, these results are beneficial for distinguish GH and PE from controls. However, there are some limitations in this study. Due to the relatively small sample size and single-center experience, the selection bias is inevitable, which limits the generalizability of the findings. As an observational cross-sectional design, the test results were measured after the onset of pregnancy induced hypertension, causality cannot be determined and the time that the examined markers change during the course of the disease was unknown. Larger and multicenter prospective studies throughout different trimesters is recommended to confirm the role of these parameters in early diagnosis and prognostic assessment for pregnancy induced hypertension. Furthermore, some unmeasured confounding factors, such as inflammatory markers should also be incorporated into future research.

## Conclusions

The peripheral blood parameters UA, ALB, LDH and MPV can be cost-efective indicators of pregnancy induced hypertension and had good diagnostic accuracy, especially for preeclampsia. Among them, UA with the highest AUC was the most important marker in identification of pregnancy induced hypertension, followed by the ALB and LDH. Our results also show that the alterations of UA, ALB and LDH are associated with both severity and pregnancy outcomes. In this regard, monitoring the alterations of the markers above during the second or third trimester of pregnacy as a supplementary, rapid, and costeffective procedure for high-risk pregnant women with pregnancy induced hypertension could provide objective evidence for appropriate interventions to prevent the progression of the disorder and improve perinatal outcomes in the country.
